# Book Reviews

**Published:** 1808-11

**Authors:** 


### CRITICAL ANALYSIS
### OF THE
### RECENT PUBLICATIONS
### ON THE
### DIFFERENT BRANCHES OF PHYSIC, SURGERY, AND MEDICAL PHILOSOPHY.

---

### *The Edinburgh Medical and Surgical Journal, No. XVI.*

ARTICLE 1—*On the Poison of Fish.* *By* C. CHISHOLM, M. D.

THIS is a very long article, and it must be admitted that the importance of the subject entitles it to all the space it occupies. Whether Dr. Chisholm is correct in the few satisfactory inferences he seems to draw, we pretend not to determine; but we must allow him merit for as much as he has done, and even as much as he has attempted. The notions of poisons are extremely prevalent among uninformed people, and especially among those who are as well fed as the inhabitants of our West India Islands. We are not here speaking of the negroes. They are not the class who lead the public opinion, nor can they always regulate their own table to their own wishes. But that hunger, which is admitted every where to be the best sauce, is on some occasions the best test concerning poisons appears by the following anecdote, selected from the paper before us.

"When any suspicion is entertained of the wholesomeness of fish, the most usual method practised in all the islands, is to boil a piece of silver, or, very often, when silver is not at hand, an onion, in the same vessel with it. If the fish is wholesome, the colour of the silver, or onion, remains unchanged; if poisonous, they become black. This is generally considered a most certain test of the quality of the fish, and its simplicity would render it the most useful. Experience has been the guide in the choice, and learned men have given their suffrage in favour of it. (Kalm's Travels in N. America, v. i. 386.) But how far we can depend on its infallibility, I shall not pretend to say, although an instance of its fallacy was related to me, by my friend Mr. Stevenson, of St. Eustatius. A Negro, of that island, purchased a barracuda, which his friends, believing to be poisonous, endeavoured to persuade him from eating. He, however, had it boiled, and to ascertain its quality, put a piece of silver into the vessel. The silver became perfectly black; but, not being disposed to lose his money, he still determined to eat the fish; he did so, and found it perfectly wholesome. Another method of detecting the poison, is to throw the heart, or the entrails, to a dog, cat, or duck, before the fish is boiled. If these prove harmless, the fish is of course course considered wholesome; on the contrary, if they prove fatal, a test of the most decided certainty is furnished of the existence of poison. But there are tests existing in the fish itself, not unfrequently, by which the poison may be detected. It is a general observation of fishermen and navigators of the channels and narrow seas among the keys or smaller islands, where fish are most abundant, that when fish of any kind, but particularly king-fish (the varieties called bastard and molatoe), Spanish mackarel (scomber cæruleo-argenteus rudus), &c. are destitute of a certain smell peculiar to them, that smell distinguished by the name "fishy smell," they ought to be considered as unwholesome. It is also remarked, by the same class of men, that when these fish are of an uncommon magnitude, they should be rejected as hurtful, or, at least, as less certainly wholesome than those of a more moderate size. The last observation is more particularly applicable to the overgrown king-fish, the barracuda, and bay and gray snapper (species of the coracinus.) In the barracuda, nature seems to be particularly careful to discover its poison by a singular distinction. When this fish is observed to have black or dark-coloured teeth, not a doubt, we are told, should remain of its poisonous property; and it is generally received as a fact, that when any of this kind of fish have proved poisonous, they have been found with black or dark-coloured teeth; and, on the other hand, that there has seldom been an instance of the white-teethed barracuda being unwholesome. Du Tertre, we have seen, has, with a different view, carefully pointed out these marks of poison.

We know not whether our readers will think with us, but we confess that after reading this paragraph, we felt considerably less interest in the whole paper. What better proof have we of the test from fishy smell, increased size, and dark coloured teeth, than from boiling with copper, or onion? Do we not know that in many parts of the old world, mackarel are considered poisonous? Lord Dover, at that time Sir Joseph York, when ambassador at the Hague, first taught the Dutch to eat this fish, which till that time, a nation containing the ablest fishermen in Europe, had rejected with horror, and to which they are scarcely yet reconciled. Among many of the less enlightened Spanish and Portuguese colonists, they are still considered in the same light. Do we not know how numerous were the supposed poisons of the ancients, and that this error was for a number of ages supported by an opinion equally erroneous, of efficacious antidotes; as even to this day we sometimes hear of the efficacy of white-witches, in counteracting the incantations of their infernal sisterhood. The plan proposed in the middle of the transcribed paragraph, is intelligible and rational. We heartily wish the paper had contained more experiments, and fewer traditions. We are, however, ready to admit, that the author has brought a subject before physicians, hitherto too much overlooked by them. We shall therefore give the substance substance of his paper, rather to induce others to enquire experimentally, than to trust to Dr. Chisholm's traditionary evidence.

In the island of Grenada, the residence of our author, during part of his official duty, he found on inquiry the poisonous fish to be "the barracuda (perca major subargentea maculata, pinnis nigrantibus of Browne, N. Hist. of Jamaica—the esox barracuda of Sloane, and becune of the French)—a species of snapper (coracinus fuscus major) gray snapper—the porgee (sparus chrysops)—the dolphin (coryphæna cæruleo varie splendens, caudâ bifurca)—the king-fish, tassard of the French, (scomber maximus, pinnulis utrinque novem, tuberculo rigido acuminato utrinque ad caudam)—the conger-eel (muræna major subolivacea)—a variety of the sprat, distinguished by the trivial name "yellow bill'd," not mentioned by Browne, (clupea thryssa, Osbeck's Faunula Sinensis)." All of these, however, are generally wholesome, excepting the last, the poisonous property of which exceeds any thing ever yet related, among all the wonders of the venomous art. "The first instance of its fatal effect I met with, was in a negro of the estate of Grand-mal, near St. George's. The poor fellow had scarcely swallowed the fish, when the most dreadful convulsions were produced, and in about half an hour he was dead. The œsophagus and stomach were in a highly inflamed state, and exhibited the appearances produced by the most active metallic poisons. When the progress of the action of the poison of this fish is less rapid, the course of symptoms is an itching all over the body, violent colicky pains, a contraction and pungent heat of the œsophagus, nausea, heat of skin, and great acceleration of pulse, giddiness, loss of sight, cold sweats, insensibility and death. The distinguishing symptoms are the contraction and pungent heat of the œsophagus. The rapidity of the action of this poison is such, that at St. Eustatius, and other of the Leeward Islands, whites and negroes have been known to expire with the sprats in their mouths still unswallowed. And yet I am informed, that at Porto Rico, the yellow-bill'd sprat is by no means poisonous, but eat with impunity—a curious circumstance, and not to be accounted for, without supposing the local existence of a marine poison of some peculiar kind."

We cannot help wishing that this case had been related more minutely, and still more, that the contents of the negroe's stomach, or other yellow-bill'd sprats of the same haul, had been offered to a dog, or some inferior animal. That we had been informed, whether the animal refused the offer, or received it with reluctance; or if forced into his œsophagus, what the consequences were. We are not surprised at meeting with no chemical analysis of these substances, well knowing how difficult, and often how unsatisfactory it is to conduct them under a tropical sun, at a distance from any apparatus, and without any partner to assist in or witness the effect of our toil.

As by this time the reader must be aware of our meaning, and as as to do justice to Dr. Chisholm, he has always acquainted us with the sources of his information, we shall content ourselves with bringing before them in as few words as possible, the contents of his paper.

The gray snapper is said to affect the bowels principally, and sometimes to produce a leprous eruption. "In the year 1786, says he, a carpenter of the name M'Arthur, his two brothers, three or four white journeymen, and some negro carpenters belonging to him, suffered severely by eating of a poisonous gray snapper. The general symptoms were such as I have described; but its mode of acting on one of the negroes is very singular, and affords perhaps an useful hint in the treatment of old ulcers. This man had had an ulcer on one of his legs, which had resisted a variety of means of cure, for more than two years; and at the time I am speaking of, it was so ill-conditioned, that amputation had been proposed as the only means of saving his life. He had eat a larger portion of the fish than his mess-mates, but at first the symptoms were similar. At the end of two days, the discharge from the ulcer became thicker, more abundant, and better coloured; but at the same time, the whole surface of his body became scored, or divided into regular squares, each score deepening into a fossula, out of which was discharged, in astonishing quantities, a substance of a thick curdly texture, and whitish colour. During six weeks, this singular discharge continued: about that period, it gradually ceased, the ulcerated surface healed; and the ulcer on his leg, in a few weeks more, no other means having been used, healed in a most perfect manner. This was literally impressing a new habit on the system, by the introduction of a more powerful and active stimulus. Instances of this do not often occur, and it may be considered as a kind of reproach to physicians, that they are almost always accidental."

We know not how to take our author, in this last dry hit on the whole profession. He cannot mean that the first experiments should be other than accidental, when he considers the subject he has to work upon, and the supposed force of the remedy: but he must be aware, that from the time when Mr. Hunter convinced the world, that the cure of syphilis by mercury could only be by inducing a stronger stimulus than the disease; this doctrine of suspending one diseased action by another, has been not only often discovered by accident, but successfully attempted, and conducted with design.

The porgee, supposed to be the sparus pargos of Foster is said to produce somewhat similar effects, but milder at least in the West India Islands.

The author does not recollect more than two instances in which the dolphin proved poisonous. In these, the disease induced readily yielded to simple remedies.

An instance is related, (we are not certain whether from our author's knowledge) in the year 1791, of a large conger, eaten by two two overseers, three stout negro men, and a negro child 3 years old, — each suffered in proportion to the quantity he consumed.

A variety of King's fisher, called the bastard, is said to be the most poisonous of this tribe, producing cholera and florid eruptions.

During the author's residence at St. Croix, he made excursions to the adjoining group of Virgin Islands. At these times, he made many inquiries concerning the poisonous fish in those seas. These are the barracuda (perca major); the yellow-billed sprat (clupea thryssa); the poisoned grooper (perca venenosa of Catesby, or trigla subfusca nebulata of Browne); the horse-eye and green-backed cavalloe (varieties, I believe, of the scomber macula nigra ad basin utriusque branchiostegæ et in utraque pinnâ pectorali); Spanish mackarel (scomber cæruleo argenteus nudus, B.); king-fish; the old wife (balistes monoceros); the hyne (coracinus minor); and some varieties of the cancer ruricola. Of these the most dangerous are the barracuda and sprat.

The information from the fishermen was uniform, as is more usually the case when a tale or tradition is repeated, than when no transaction is related by any number of people. All agreed in supposing copper to be the source of the poison; though they consider their feeding on sea moss, (corallina opuntia) during the spawning season as a cause. Might it not be questioned, whether a change in their secretions during this important season, may not render them poisonous for their own protection? By well attested records, it seems certain that the yellow-billed sprats are wholesome on the north-side of the island, and it seems scarcely possible to doubt that they are poisonous in some shoals to which they resort for the propagation of the species. So poisonous, that instances were related of negroes dying in the very act of swallowing the fish. Nay a physician of eminence at Christianstadt, related to our author an instance of a negro, who, informed of his danger, had spit the fish out of his mouth before swallowing any part, yet died in consequence of mastication only. If this were really the cause of the negroe's death, it is impossible to account for it by any cupreous impregnation.

The author next proceeds to the result of his own immediate inquiry, which he admits is less satisfactory.

"The inhabitants of the West India Islands, in their opinion respecting this poison, may be divided into those, 1. Who believe copper to be the basis of it; 2. Those who attribute all its pernicious qualities to the galere or gally-fish (medusæ and holothuriæ), which are supposed to constitute the food of those fish which possess it; and 3. The few who assign the poison to the manchineel apple, and probably as it relates to the varietes of land crab, found poisonous, they are right."

The most prevailing opinion is, that copper is the source of the poison. To discover the source of this metal, our author was at some some pains, and we hope his researches will hereafter furnish the mother country or the Islanders with a new source of wealth in this valuable metal.

"The submarine ridge or bank, extending from Grenada to Tobago, called by the fishermen of the former island "Copper Bank," is, I suspect, a vein of the mountain-green, and may be, there are good grounds for believing, an extension of that demonstrated in the leewaid Caribbean Sea. Nor is it at all improbable that it might be discovered among the Bahama Islands, were a careful investigation made. The poisonous quality of the fish of the Bahama Seas has been noted and experienced from the days of Catesby to the present. In tracing the tract marked by the presence of poisonous fish, it will be considered a circumstance of at least curious importance, that at Saba, Sombnero, Dog, and Pear, &c. forming a kind of sweeping line nearly N. W. the fish are as poisonous as at St. Eustatius and Sandy point; whilst, to the westward of this line, at St. Thomas, St. John, Tortola, Virgin Gorda, Crab and Passage Islands, and Porto Rico, and to the eastward at St. Martin's, Anguilla, Anegada, they are always perfectly wholesome. St. Bartholomew is, however, an exception, although closely adjoining St. Martin's."

This geographical sketch is carried on much further by Dr. Chisholm; but we shall attend to the arguments in favour of the probability of poison from this source. A British frigate was wrecked on one of the Virgin Islands; oysters grew on her copper bottom, which were eaten not without producing cholera, and excruciating tormina, though in no instance fatal.—The account given by Dr. Blane, of the case of an officer and two seamen poisoned by copper vessels is shown to be similar to the poison from these fish, Fourcroy assures us that oxyds of copper are more virulent than of any other metal, and it is remarked that the muriatic acid in this part of the world, increased by heat, is a powerful solvent of copper. Many other arguments are brought, in confirmation of the probability that this poison is of a cupreous origin.

Dr. Chisholm next considers whether the poison of some fish may arise from the manchineel apple, which is in some places supposed to be their food. After noticing the authority of several French writers, he doubts whether such a cause is sufficient, or whether any but some of the testaceous tribe make it their food. His own observations, as well as those of the inhabitants, and of several writers, would show that the manchineel apple is itself not poisonous. "The solution of the following question says he, may lead to interesting results. If fish are highly susceptible of certain vegetable poisons, why does not the same effect result from the natural application of the poison, which is the result of our present enquiry?" Instances are produced, in which fish are stupefied, or killed by vegetable poisons, yet are afterwards wholesome food. Hence he concludes, that supposing the fish even poisoned by manchineel manchineel apple, yet we might expect they might be afterwards eaten with safety.

The opinion which chiefly prevails among the French writers is, that the galere or medusa is the cause of the poison of fish. But this the author proves to be at least problematical, by the number of places in which the medusa is found, but which abound with wholesome fish. It is even doubted by Dr. C. whether the fish medusa itself is as venomous as is generally believed, yet he seems to give more credit to some wonderful traditions concerning it, than his doubts would authorize.

We shall not follow our author through all his remarks and conjectures concerning the probability of an animal being supported by a substance which renders him poisonous when taken as food, nor into an illustration of the same by Gortanner's law of irritability, nor the many other instances of poisons which are produced, many of them, in our opinion, on very uncertain authority. The numbers, as he observes, ought to have been greatly multiplied, but he wishes to confine himself to a few *classical instances.* These kind of references are certainly very interesting, but can we call them satisfactory? What, though the story of Mithridates is related by Justin, and admitted by the modern compilers of the history of Pontus.—What, though Galen admits it also, and believes that a similar habit had been impressed on the Emperor Aurelian by himself.—Are the authorities of those days sufficient to induce a belief of a power, which, if well established, would have been perpetually referred to? Or, is there any greater probability of the story concerning the Sultan of Cambaia than of the Arabian Knight?

Uncertain as we are of the reality of some of these poisons, which the author did not witness himself, we shall be very short on the subject of the cure from the effect of them. Against those which poison by mastication, there can be but little chance of success by any remedy. The principal antidote against the rest is salt. On this, and the rest of the paper, we shall forbear to make any further remarks, only repeating our wishes that the subject may be taken up experimentally by those who have such opportunities.

Article 2.—*Medical Reports for Nottingham, from March* 1807, *to March* 1808.

The author, after a few judicious remarks, begins his account with an examination of the epidemic constitution from April 1807 to March 1808.

In April, pulmonary complaints appeared, as are usual at these seasons, but were easily removed; a contagious porrigo, or scalded head appeared, and continued to be very troublesome.

The most remarkable thing in the two subsequent months, is, that rubeola, which was so fatal in London, was attended with little danger, though epidemic, in Northampton. In August, however, it became very serious, and acquired, as the author remarks, a disposition to gangrene. In October, however, it again became milder. During the same month typhus was attended with diarrhœa; the principal remedy was cold sponging with vinegar and water, and the exhibition of nitric acid. Wine was found injurious till perspiration appeared.

In November, catarrh took place of measles, and remittent fevers of children were very prevalent. Synochus among grown people. Typhus was for the most part mild, but often required purgatives, and was constantly the worse for astringent remedies, when diarrhœa spontaneously supervened.

In December catarrh was so general, though for the most part mild, as to acquire the name of influenza, yet infants and convalescents from measles and other diseases, fell victims to it. In January the catarrh continued, with pain in the side, which yielded to blisters, but those who were bled recovered very slowly.

The present alarm concerning rabies, induces us to transcribe the following account of an epizootic in the canine species.

"Much alarm has been excited within the last two months in this town and neighbourhood by a disease among dogs, said to resemble rabies; several men and children were bit, but no hydrophobia has ensued, although few employed any remedy. Some certainly went to the coast for sea-bathing, which has had the merit of being considered a certain preventive, but experience has too often proved the fallacy of relying on this plan. A boy was admitted into the hospital with a wound on the fleshy part of the thumb from the bite of a dog supposed mad. Caustic was applied to the part, and small doses of calomel exhibited night and morning; the injury had been received more than seven days before he applied for assistance, but no symptom of disease appeared, and the boy has ever since remained in good health. The dog was destroyed as soon as possible, but not before he had bit a pig, which, after the lapse of some days, was killed from a suspicion of madness.

"When the cry of 'mad-dog, mad-dog,' arises, death is the inevitable portion of the animal; and, by the vulgar, it would be considered the highest point of rashness to attempt saving it from immediate destruction; but one instance has occurred, where a man had sufficient firmness to resist the public cry, and to put his dog, which had bit two children, into a place of safety; he eat and drank the same day as usual; this a rabid animal might have done; but he has continued ever since in perfect health; nor have the children, although no means were employed, experienced one unpleasant symptom: had this animal, on the contrary, been destroyed, it would have been added to the list of mad-dogs, and the relations of the children would have remained, at this moment, in a state of suspence.

There certainly has been considerable disease among dogs, attended with different symptoms; some were suddenly attacked with giddiness, turning round several times, run staggering and biting at objects in their way, and then unexpectedly drop down dead; dead; and this probably in two or three hours after the first seizure. One looked dull in the eye, and frothed in the mouth; a vomit of tartrit of antimony cleared his stomach and bowels, and removed all disease. These complaints were most prevalent in dogs exposed to the temporary influence of a fire. In the public newspapers, about this time, a circumstance is related of a strange dog entering a coffee-room, on a very cold frosty day, and lying down before the fire, from whence, after sleeping some time, he sprung up, and made repeated attempts to bite those in the room, until destroyed; this might be rabies; but it may, with equal probability, be attributed to frenitis, arising from the sudden alteration of temperature, to which the dog exposed himself.

"In addition to the instances of disease in the dog which have been related, two others occurred in June 1808. A small dog appeared dull for a day, then bit his master in the hand, and ran away. He was cautiously pursued, brought home, and placed in a state of confinement, where, after refusing all food, he died on the second day. The body being opened, the stomach, œsophagus, and intestines, were free from any morbid appearance. The lungs were much inflamed. Caustic was applied to the bitten part soon after the wound had been received; about a week after, the man complained of nausea, and oppression at the præcordia; an emetic was exhibited, and assisted in its operation by warm water, with a few drops of the water of ammonia, since which he has continued in perfect health. The other instance certainly presents a very unfavourable appearance; and, as the reporter paid particular attention to the symptoms which the animal exhibited, he thinks a full detail may be acceptable; and he is the more disposed to this, from the little information which he has been able to procure on this subject from books, or personal inquiires.

"The dog, a terrier, was reported to have been bit by a strange dog, reputed mad, that ran through the town, on the 6th of June, and in its way bit other animals, all of whom were immediately destroyed; but as the report of this dog being injured was not conveyed to the owner, he was allowed his liberty as usual. About 16 days after, he was observed repeatedly quarrelling with a large powerful mastiff, on the same premises, with whom he had always been friendly; this circumstance did not excite any surprise at the time, but was supposed to arise from both dogs being more confined than usual, in compliance with a public order for the confinement of dogs during the prevalence of rabies. On Friday evening, July 1st, he followed his master's daughter and her playmate into the hay field, where, during their play, the little girl fell upon the hay near him; he immediately bit her in the leg; but being naturally a savage animal, and the bite slight, it was attributed to accidental surprise, and no notice taken of the dog, until the next day, when he was observed to look particularly dull in the eye, to hold the mouth wide open, with the lower jaw fallen; a disinclination to food, although he eat a small portion of buttered

 bread.

bread. These appearances exciting strong suspicion of rabies, he was immediately chained up in his kennel. On the evening of Saturday July 2d the reporter first saw him, and he will now attempt to give a clear detail of the result.

"The eyes were dull, and almost closed by a yellowish matter; the mouth half open, the lower jaw fallen, and only closed when crouching upon the ground; vapour constantly issuing from the mouth; makes many vain attempts to pass urine and fæces; scratches up the earth with his fore-feet, biting it, and then rolling on it; lies down quietly for a few seconds, then starts up suddenly, and howls in a very unnatural tone; the tail hangs down, ears are erect, does not shew any fear, has not swallowed food since the morning; he appears rational, advances to the person calling him, as far as the chain will permit: water being poured into a bason before him, he rushes towards it, and eagerly laps, but makes no attempts to swallow; takes buttered bread in the mouth, turns it round, and lets it fall out again; keeps the tongue, which is quite black, entirely within the mouth; no flow of saliva, but, on the contrary, the mouth appears parched; makes no attempt to bite the kennel; shews great dislike to be left alone, by horrid howling.

"July 3d—Has howled almost the whole night, has not swallowed food, laps water with the same eagerness as yesterday, has not passed any urine or fæces, has made several ineffectual attempts to vomit, much more restless and impatient, mouth as yesterday, no flow of saliva; gnaws the kennel, bites the chain, or any thing that appears to confine him.

"July 4th—Bites at any object put near him; does not refuse food, and still laps water, but cannot swallow; mouth as before. In the afternoon some castor oil was poured down his throat by a strong man, who, for safety, had fixed his head by a fork, placed over his neck, and forced into the ground at the extremity of his chain; he did not attempt to bite, but became more tractable; swallowed some gruel, vomited much dark-coloured fluid, passed both urine and fæces, became more composed, but was found dead at four the next morning. The body was opened as soon as possible by Mr. Kenney, veterinary surgeon of this town. Much slimy mucus was found under the tongue; the parotid glands greatly enlarged, the uvula and inner coat of the stomach much inflamed, one lung was also inflamed, the stomach contained some dark-coloured fluid, some coagulated blood between the dura mater and pia mater, but no inflammation of the membranes; the vessels of the brain turgid with blood, the jaws free from any bruise or injury, no inflammation of the œsophagus, or intestines, which were quite empty; the bladder was free from disease.

"It yet remains doubtful if the disease of which this animal died be rabies; very little satisfactory detail of the symptoms in the dog is to be met with, and dissections are still more rare. The clearest statement of the disease is from the pen of Mr. Meynel, whose whose opportunities for information on this subject cannot be disputed: he describes a disease called the dumb madness, which bears strong analogy to the one detailed here. Dr. Hamilton of Ipswich has, ten years since, given to the public two volumes on hydrophobia, which contain much valuable matter. The young girl who was bit had caustic applied to the part, and in about six days after was sent to the coast for sea-bathing, where the surgeon extirpated the wounded part with the knife.

"No satisfactory information could be procured from those persons in this neighbourhood who have been brought up to the care of hounds; they all judged by the eye, and were incapable of conveying their information but in a mass of contradiction, from which it is difficult to draw the truth."

In February the catarrh became almost universal, and generally required blood-letting with some freedom. Such was the case, with measles, which now appeared principally among adults. — The account closes with the description of an irregular intermittent in some soldiers who had arrived from a marshy quarter. The disease, as Dr. T. observes, was so universal as to merit a full detail.

"The type was very irregular; six, seven, ten days, or more, elapsed between the paroxysms, which came on irregularly, with very strong rigour, followed by great heat and much sweating; tongue of a lardish white fur, sometimes yellowish appearance; pulse full, hard, and quick; countenance tinged with yellow. The rigour returned with some in the evening, but with others in the morning. Blood drawn exhibited a buffy coat. The patients were all impressed with the idea that the disease was ague. Bark increased the fever, and was, in some cases, followed by violent hæmorrhages from the nostrils. Febrifuge medicines, bleeding, purgatives, all were tried in vain, with the severe cases; some of the slighter were cured under one or other of these plans of treatment. In a few, cold affusion was employed in the hot fit; it shortened the paroxysms considerably, but did not produce any farther alleviation. Arsenic was then given; but as those who took no medicine were gradually recovering, it was omitted, and the troops were marched from this place before the result of all the cases could be known. Although in no one instance did the disease end fatally, yet it is also probable that Nature effected the cure. Home gives an account of a fever among the troops in Germany, which bears a strong resemblance to this disease."

A meteorological table follows, well constructed, and a table of diseases.

Art. 3. — *Cases of Bronchial Polypi, with Introductory Remarks.* By John Cheyne, M. D.

The author divides bronchial polypi into two species. The first is a mere coagulation of the blood under hæmoptoe. The others, he conceives, are symptomatic of a more chronic disease. "They are," he observes, "preceded by catarrhal complaints,

 and and attended with cough, wheezing, and dyspnœa. The fit of coughing which displaces them is sometimes alarmingly violent."

"Bronchial polypi, of the second kind, differ materially from those first described, which were said to be mere coagula, separated from the blood which had been poured into the lungs. Those of the second species are produced by a secreting surface, in a state of inflammation. They are justly held as analogous to the membrane of croup. Yet, the action which produces bronchial polypus never rises to such a height as in croup. This view of the disease leads to the cure. The consideration of this need not detain me; we have only to adept the general indications laid down for the treatment of inflammations to the disease in question."

We have given the passage in the author's own words, not having the same aptitude at assigning causes for effect.

Art. 4.—*On the Sensibility of the Inflamed Cornea to the Transmission of Light.* By John Vetch, M. D. in charge of the Ophthalmia Depôt, Selsey Barracks.

The ingenious author of this paper begins by remarking, that all the organs of sense under inflammation are on some occasions subject to greater, and in others to less sensibility from this change. This he observes is applicable to the eye in its sensibility to light under inflammation. In the violent stage of the Egyptian Ophthalmia, he remarks the eye was less susceptible of light, but in the milder, the susceptibility amounts almost to an *intolerantia lucis.* The form of the disease may be early discovered. In the more violent the vessels are exceedingly minute, the formation of pus more abundant, and there is an early tendency to tumefaction in the conjunctiva. The chemosis which succeeds is not formed by a plexus of vessels, but by an effusion which may be compared to a ring of coral surrounding the cornea, varying in tinges like that substance. As the deeper parts of the eye become affected, it is rendered less and less sensible of light, a discharge of matter follows, and the swelling of the palpebræ precludes all further inspection.

When the vessels, on the contrary, are larger, more distinct and tortuous, the disease is usually confined to the conjunctiva, from which however it does not readily recede. The vessels, from the beginning, extend over the whole surface, and entrench on the margin of the cornea, which may gradually lose its transparences, and thus prevent the return of future vision.

The paper contains some other very judicious remarks, and concludes with the following hints as to cure.

"In farther illustration of the subject, and without entering into any detail of the treatment, I may add, that the irritability arising from the affection of the cornea, by the encroachment of the *conjunctiva*, is best obviated by a well regulated application of the *argent. nitrat.* as near as possible to the margin which has become affected. The use of this remedy I first derived from Mr. Peach, surgeon to the 2d battalion 52d regiment, who then employed employed it for the destruction of vessels in chronic cases of opacity. I have been lately informed by Mr. Draper, surgeon to the 2d battalion 78th regiment, that he employed the same remedy very extensively in Egypt. It is one of much utility in all chronic cases of ophthalmia, and, in most instances, may be substituted with advantage in the room of scarification, which, in the ophthalmia propagated by infection, I have always found strong reason to reprobate."

A Postscript is subjoined, containing some Remarks on Mr. Ware's late Tract. These we shall reserve till that performance comes before us.

Article 5.—*Journal of the State of the Sick on board one of his Majesty's (line of Battle) Ships, during a Cruize to and from the West Indies, from the* 18*th of January to the* 18*th of April* 1808. By J. F. Assistant Surgeon.

Article 6.—*Observations on Yellow Fever.* By D. J. H. Dickson, M. D.

We are glad to find so judicious an author, who has had large practical opportunities, confirm an opinion we have uniformly supported, that the few instances recorded of contagion in yellow fever are to be imputed to typhus occurring in those regions, and assuming the appearance of the endemic fever. In that light our author considers the yellow fever, though attacking strangers in a peculiar manner, till they become habituated to the climate. This induces him to differ from those writers, who support an analogy between this fever and plague, both of which Dr. D. has had an opportunity of seeing.

Article 7.—*Cases of Gout in Negroes.* By Mr. D. Quarrier, Surgeon of the Princess Charlotte.

Article 8.—*Biographical Sketch of* Dr. Darwin.

This, of course, contains little more than the history of Dr. D's studies and publications.

The Inquirer directs us to the search of the Cause of the Sweating Sickness.

This inquiry was suggested by the last Number of Dr. Willan's "Diseases of the Skin." In our Remarks on that Work, we purposely omitted noticing what appeared not only an unfounded opinion, but unnecessarily introduced in an account of cutaneous diseases. But, if we were dissatisfied with Dr. Willan's solitary hint, we have derived no more satisfaction from the Inquirer's longer Essay.

It is first remarked, in contradiction to Dr. Caius's observation, that the disease could not be confined to the rich, as 980 persons died within a few days in Shrewsbury. There is, however, a material difference between a disease which attacks principally the rich, and one which is chiefly confined to the poor; nor is it possible, that Dr. Caius could have been mistaken in the grand outlines. Another thing is admitted by the Inquirer, contrary to

 Dr. Caius,

Dr. Caius and every medical account that we have seen ; namely, that the disease was contagious.

It is unnecessary to take notice of the numerous arguments brought by the Inquirer, to prove that the disease did not arise from damaged corn, as we know not a single circumstance which could be produced to favour such an opinion ; but it is very remarkable that it should be questioned, whether typhus fever is not a common attendant on times of scarcity or distress.

Is not the coincidence of λοιμος και λιμος almost coeval with any authentic accounts of history. The inhabitants of the southern metropolis cannot but feel flattered at the value placed on their Institutions; but a regard to truth, and to a just chronicle of events, obliges us to remark, that so little has typhus fever prevailed, as to render it unnecessary to increase the boundaries of the Fever house, which cannot with any safety contain more than half a score patients with their necessary attendants. Far be it from us to undervalue the benevolent designs of the governors, or the industry of the medical officers. Should necessity require larger accommodations, doubtless the same encouragement will be increased ; and as we have followed the example of Manchester and Liverpool, so we shall keep pace with them. But it cannot be doubted that we owe our exemption from typhus to the meliorated condition of the labouring class. During the year of scarcity, the price of labour was increased, and that price has not diminished as provisions have become cheaper. During the short interval of peace, our builders reassumed their activity, and the return of war has not materially interrupted them.

We are glad to find among the Articles of Intelligence, that his Majesty has been pleased to double the pension of that meritorious officer Dr. Gilbert Blane, in compensation for his having been deprived of his office of Commissioner to the Sick and Wounded, on the reduction of that board after the peace of Amiens.

---

*Practical Electricity, and Galvanism, containing a Series of Experiments calculated for the Use of those who are desirous of becoming acquainted with that Branch of Science; illustrated with Nine Copper Plates.* By John Cuthbertson, Philosophical Instrument Maker, and Fellow of the Philosohpical Societies of Holland and Utrecht.

Not only has Galvanism added a new wing to the Electrical battery, but even the Electrical apparatus itself has received considerable alterations within these few years. Its application to medicine has also greatly increased ; nor is it possible to say what may be accomplished, when experiments are sufficiently multiplied, to explain the great variety of its effects produced on different subjects.

Nothing can be more judicious than the compilation before us at such a time. The simplicity of the language, the easy induction to the most abstruse parts hitherto discovered in this interesting ing science, and the lively illustration by such numerous and well executed plates, render this work a most complete introduction for those, whose distance from the larger towns, or other avocations, prevent their attendance on a regular course of lectures.

Of a work of this kind it is impossible to do much more than announce the plan and general contents. In a preface we are informed, that the author first began to publish in Holland, where electricity was imperfectly known and among very few. His success in rendering the science more popular, induced him to enlarge his work by new experiments, and to add something to the Franklinian Theory. On this we shall transcribe the author's own words, and also add the conclusion of his preface, as descriptive of the object of this work.

"With regard to the theory, which I have advanced in Part 1, it will be found to coincide with that of Franklin; but whether or no it is the true theory I do not pretend to determine. I can only say, that all the experiments with which I am acquainted can be more easily explained by it than by any other.

"I have endeavoured to arrange and methodize the experiments so as to render them easy and pleasing to the young practitioner, in hopes that it will lead fresh admirers into a walk where such wide scenes of interest and amusement are continually unfolding themselves. I shall conclude with observing, that in publishing the present Treatise in my native country I feel considerable diffidence, arising from the consciousness that my language in many parts, from having bee nso long accustomed to speak a foreign tongue, may offend the ear of my reader, However, I will venture to throw myself on his liberality; and in soliciting his indulgence, I farther plead my occupation as an artist, and the various interruptions I must have experienced from that scource, in defence of any other errors which may be perceived."

The contents of the work comprehend all that was known of the science before the discoveries made by Professor Davy, and these are readily understood by those who make themselves acquainted with the laws of Galvanism.

We shall offer a single passage, which we have thought could be most easily selected, to show the general style of the author.

"*To direct a Galvanic Shock, or pass the galvanic fluid through any particular part of the body, for medical purposes.*

"Exp. 199. Suppose a person is to be galvanized through the head; place the patient and trough as is represented in fig. 104: the interstices of the trough being filled with diluted nitrous acid, put one end of each director in the holes at the end of the trough, the other ends being directed to the place where the galvanic fluid is intended to act;—having previously screwed to the ends of the directors two button-like pieces of metal, covered with cloth, which must be well soaked in the same liquid as is used for the trough, hold them to the patient, as is represented in in the figure, and press them gently to the part. On the first application the patient will feel a shock, and see a flash of light—if the ends of the directors are not removed the patient will experience no more shocks; but a current of galvanic fluid will run through the head, entering in from that end of the director which is annexed to the zinc end of the trough, and passing out to the copper end, by the other director; this current will be felt, or not, by the patient, according to the size of the trough, or number of plates. If it is desired that the patient should have shocks, one of the directors must only touch the patient by intervals; the other being kept close to the part, each touch will produce a shock. In the above method the power of the whole trough is felt—if it should be too severe, screw on to the end of either of the directors the spring clasp c, and instead of fixing it to the end of the trough, slip it on any of the plates, and the shock will be moderated, according to the number of plates included by the directors.

"It is the shock given in different directions, which produces muscular motion in animals, after animation is suspended, from whence it was supposed it might be of use in resuscitation under such circumstances, &c. This I have tried on two malefactors, but without success.

"The apparatus used for the above purpose, consisted of three troughs combined together, each of which contained forty plates of zinc and copper of about one inch and a quarter square surface. When one of the conductors was applied to the mouth and the other to the ear, the jaws began to quiver, the adjoining muscles were horribly contorted, and the left eye actually opened. (For a farther account of these experiments, *see an Account of Galvanism by John Aldini, printed for Cuthell and Martin, London,* 1803.) In this work the writer says, that the effects produced by galvanism on the human frame are different from those communicated by electrical machines. This he has not proved; it is certain that muscular motion can be produced by common electricity, as powerfully as by galvanism, but not in such quick succession, because when muscular motion is to be produced by common electricity, it must be by discharges, either from coated glass or a very large insulated conductor, which takes some time in charging; more or less depending upon the power of the electrical machine; indeed, there has not yet been made an electrical machine that can throw off so much electric fluid, in a given time, as can be produced by a galvanic trough of only thirty-two pairs of plates. The latter is capable of igniting eight inches of iron wire, with the same power as two highly charged jars, and the experiment can be repeated several times by the latter, before the jars can be charged to such a degree, as to produce the same effect once. Galvanism has also the advantage over common electricity, that it can be administered in all circumstances where common electricity can; and in some circumstances, where common electricity cannot; it is therefore very much to be lamented that something has not yet been set on foot, in order to give it a fair trial in cases of suspended animation."

